# Predicting the Post-therapy Severity Level (UPDRS-III) of Patients With Parkinson's Disease After Drug Therapy by Using the Dynamic Connectivity Efficiency of fMRI

**DOI:** 10.3389/fneur.2019.00668

**Published:** 2019-07-02

**Authors:** Xuesong Li, Yuhui Xiong, Simin Liu, Rongsong Zhou, Zhangxuan Hu, Yan Tong, Le He, Zhendong Niu, Yu Ma, Hua Guo

**Affiliations:** ^1^School of Computer Science and Technology, Beijing Institute of Technology, Beijing, China; ^2^Department of Biomedical Engineering, Center for Biomedical Imaging Research, School of Medicine, Tsinghua University, Beijing, China; ^3^Department of Neurosurgery, Tsinghua University Yuquan Hospital, Beijing, China; ^4^Nuffield Department of Clinical Neurosciences, Wellcome Centre for Integrative Neuroimaging, FMRIB, University of Oxford, Oxford, United Kingdom

**Keywords:** fMRI, dynamic nodal efficiency, Parkinson's disease, drug treatment, prediction of post-therapy severity level

## Abstract

Parkinson's disease (PD) is a multi-systemic disease in the brain arising from the dysfunction of several neural networks. The diagnosis and treatment of PD have gained more attention for clinical researchers. While there have been many fMRI studies about functional topological changes of PD patients, whether the dynamic changes of functional connectivity can predict the drug therapy effect is still unclear. The primary objective of this study was to assess whether large-scale functional efficiency changes of topological network are detectable in PD patients, and to explore whether the severity level (UPDRS-III) after drug treatment can be predicted by the pre-treatment resting-state fMRI (rs-fMRI). Here, we recruited 62 Parkinson's disease patients and calculated the dynamic nodal efficiency networks based on rs-fMRI. With connectome-based predictive models using the least absolute shrinkage and selection operator, we demonstrated that the dynamic nodal efficiency properties predict drug therapy effect well. The contributed regions for the prediction include hippocampus, post-central gyrus, cingulate gyrus, and orbital gyrus. Specifically, the connections between hippocampus and cingulate gyrus, hippocampus and insular gyrus, insular gyrus, and orbital gyrus are positively related to the recovery (post-therapy severity level) after drug therapy. The analysis of these connection features may provide important information for clinical treatment of PD patients.

## Introduction

Parkinson's disease (PD) is one of the most common neurodegenerative disorders. It is clinically characterized by some specific motor symptoms, including rigidity, slowness of movement, tremor at rest, bradykinesia, and postural instability and some other non-motor symptoms such as cognitive deficits, impaired olfaction, emotional problems (1, 2). PD can be considered as a multi-systemic disease in the brain arising from dysfunction in several neural networks (3–5). The motor and cognitive impairments in PD have been related to abnormal functional connectivity and disrupted network integration in the brain (6–8).

Several graph theoretic studies revealed an abnormal topological organization of functional brain networks in PD patients. Specifically, Skidmore et al. combined fMRI and graph analysis to find a smaller global efficiency of brain networks in advanced PD patients (9). Wei et al. found that PD had significantly decreased efficiency in the cortico-basal ganglia motor pathway (10). In addition, Dubbelink et al. using magnetoencephalography and graph theory, reported that impaired local network efficiency and network decentralization are very early features of PD that continue to progress over time, along with reductions in global efficiency (6). In summary, the graph theory provides a powerful and general framework to characterize brain connectivity at global and local levels, and offers a collection of metrics that can quantify the segregation and integration of information within functional networks among the brain regions. However, most of the previous studies did not consider the important dynamic properties of FC over time, such as the dynamic nodal efficiency; instead, FC was usually assumed to be constant during the rs-fMRI experiment (8).

The graph theory-based approach applied to dynamic FC show that the variability in brain network may also provide important information on the underlying nature of neurodegeneration. In a study by Yu et.al., the reduced variability of local and global network efficiency was detected in a patient with schizophrenia (11). In a more recent PD study, dynamic topological properties of brain networks can characterize the underlying nature of Parkinson's disease and correlate with clinical features (8).

The dynamic property of fMRI can enrich the graph theory. We wonder if the dynamic nodal efficiency (dnE) can be used to predict the recovery effect after drug therapy (i.e., post-therapy severity level) of PD patients. If possible, it may provide useful guidance information for drug therapy. Connectome-based predictive modeling is a recently developed data-driven method for identifying the relationship between functional brain connectivity and the behavioral and cognitive variables of interest, and then predicting the behavior of patients (12–14). The predictive modeling procedure has been applied to analyze connectivity, such as attention control and temperament trait (14, 15). Its core idea is the cooperative analysis of the relationship between behavior and FC, finally finding the strong functional networks that are correlated to the behavior with statistical significance. It provides an effective way to explore the correlation between altered topological properties and clinical indexes of interest.

In the present study, we used rs-fMRI and sliding-window analysis to build the individual dnE network by computing each nodal efficiency of each sliding-window and predicted the post-therapy severity level of PD patients. The global efficiency is chosen to calculate the dnE, since it may reveal more PD properties than local efficiency, as indicated by Kim et al. whose study showed a significant difference in global efficiency between PD and the healthy control, but not in local efficiency (8). Specifically, we proposed a rigorous cross-validated prediction framework incorporating feature selection and regression techniques, to predict the drug therapy effect of levodopa (the most commonly used drug in PD treatment), which is evaluated by Unified Parkinson Disease Rating Scale III (UPDRS-III) (16) scores, using the rs-fMRI data from 62 PD patients. We aim to investigate the possibility of predicting individual after-therapy UPDRS-III scores using whole-brain dnE network. The post-therapy UPDRS-III scores for certain patients was estimated, and the potentially important connections that contribute to the recovery degree were predicted by the rs-fMRI data.

## Materials and Methods

### Subjects

Sixty-two subjects (mean age, 58.5 ± 10.1 years; 31 females and 31 male patients) were recruited from Tsinghua University Yuquan Hospital, Beijing, China. Patients were told to stop taking drugs 12 h before the rs-fMRI scan (before therapy). Patients diagnosed with PD based on the UK Brain Bank criteria (17) were enrolled. Exclusion criteria includes a history of psychiatric or neurological disease other than PD, other major medical diseases, head injury, alcohol/drug dependency/abuse (8). Disease severity of each patient was evaluated by the UPDRS-III (16) scores given by an experienced specialist after taking levodopa, including the medication-on and medication-off states. These PD patients took different doses of levodopa, according to a widely used guidance (18) for each patient. None of them have taken other medicines. Details of the demographic information can be found in Table 1. All participants signed the informed consent form before the experiment. This research was approved by the Ethics Committee of Tsinghua University Yuquan Hospital.

**Table 1:** Participant demographic and clinical characteristics.

	**Mean(±SD)**
Age (years)	58.5(±10.1)
Disease duration (years)	10.4(±4.4)
MoCA	21.6(±5.5)
Depression score (BDI-II)	8.5(±10.0)
Levodopa equivalent daily dose (mg)	720.4(±295.7)
Hoehn and Yahr stage	3.7(±0.6)
Frame-wise displacement (mm)	0.33±(0.20)
Medication-off UPDRS-III	44.1(±12.0)
Medication-on UPDRS-III	22.2(±11.8)

*Values are given as mean and SD. MoCA, Montreal Cognitive Assessment; BDI-II, Beck Depression Inventory-II; UPDRS-III, Unified Parkinson Disease Rating Scale III*.

## MR Image Acquisition

All data were collected on a 3T Philips Achieva MRI scanner (Philips Healthcare, Best, The Netherlands) with a 32-channel head coil. Head motion was controlled by fixing the head during scanning. Resting-state blood-oxygenation-level dependent (BOLD) signals were collected with following imaging parameters: 35 axial slices; repetition time (TR) = 2,000 ms; echo time (TE) = 30 ms; flip angle (FA) = 90°; slice thickness = 4.0 mm; slice gap = 0.8 mm; acquisition matrix = 64 × 64; field of view = 224 × 224 mm^2^. All the PD patients have only experienced one rs-fMRI scan, which was carried out before taking levodopa. During the scan, the participants were instructed to keep their eyes closed, relax their minds, and remain as motionless as possible but not to fall asleep. The rs-fMRI scan with 240 dynamic scans lasted for 8 min. High-resolution T1-weighted structural images in coronal view were acquired with slice thickness of 1 mm without slice gap. Other sequence parameters were: TR/TE = 7.64/3.73 ms, FOV = 256 × 256 mm^2^ (acquisition matrix = 256 × 256 × 160).

### Data Processing and Network Analysis

The pre-processing of rs-fMRI data was conducted using the SPM12 (http://www.fil.ion.ucl.ac.uk/spm) and GRETNA (19) software. The first four scans were discarded to allow for magnetization equilibration. Four subjects with the mean frame-wise displacement value exceeding the maximum displacement of 1 mm were excluded from either the above demographic information or subsequent data analysis. Data were realigned to the first volume to correct for head movement. A 0.01–0.10 Hz band-pass was used to reduce the effects of low frequency drift and high-frequency physiological noises. The nuisance signal regression (24-parameter head motion profiles, global signal, CSF signal, and WM signal) was performed. Data were spatially smoothed with a 4 mm full-width at half-maximum Gaussian kernel. In order to perform group analysis, the first scan of fMRI time series was co-registered to the same participant's T1-weighted images. The transformed T1 structural images were normalized to the Montreal Neurological Institute (MNI) template space, using the voxel size of 3 × 3 × 3 mm^3^.

**Figure 1:**
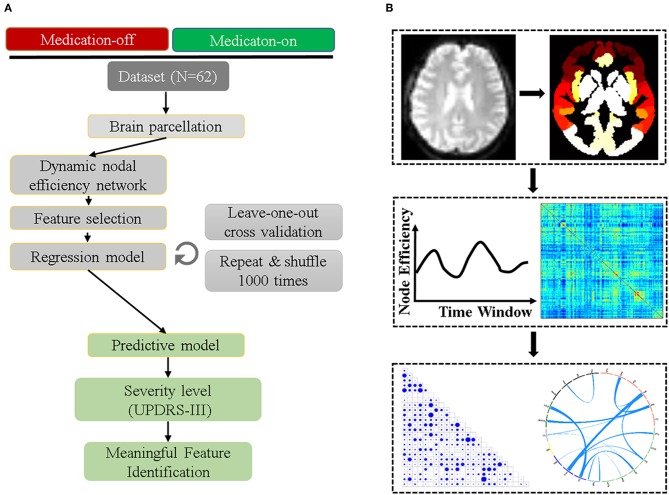
The prediction and validation flowchart incorporating feature selection and regression analysis. **(A)** shows the detailed steps of the data preprocessing including parcellation, efficiency network computing, feature selection, regression model, and the final feature verification. **(B)** is the related information from the image preprocessing to feature identified.

The flowchart of the subsequent data processing is shown in Figure 1. The GRETNA software was used to construct the whole-brain networks for each sliding-window (19). The human Brainnetome Atlas (http://atlas.brainnetome.org/) was applied to obtain 246 brain regions (i.e., nodes, with 123 in each hemisphere), including 210 cortical and 36 subcortical regions (20). The sliding-window approach was used to explore the time-varying changes of FC. The window was slided by 2 s along the 240 dyanmic scans (480 s). We chose the window size of 50 time points for the trade-off between the accuracy of capturing state transitions accurately and the number of overall state transitions (21), resulting in 191 consecutive windows across the entire scan. For each sliding window for a participant, the nodal efficiency was computed, resulting in a 191 nodal efficiency curve. For each patient, the dnE matrix (246 × 246) were calculated by computing the inter-node pearson correlation of the 191-time-point dynamic efficiency curve. The value of each element in the dynamic nodeal efficiency matrix ranges between −1 and 1.

### Prediction Model

The least absolute shrinkage and selection operator (LASSO) method (14) was performed to select the features and build the model.

The least absolute shrinkage and selection operator (LASSO) is a regression analysis method that performs both variable selection and regularization in order to enhance the prediction accuracy and interpretability of the statistical model it produces. The object function is as below:

$$\underset{\beta }{\min}{\left\|y-\text{x}\beta \right\|}_{2}^{2}+\frac{1}{2}\lambda {\left\|\beta \right\|}_{1}$$

where **x** denotes the dnE matrix and **y** donates the actual UPDRS-III scores. The objective of the whole regression process is to solve the matrix **β** by minimizing the loss function. This L1-norm regularization typically sets most coefficients to zero and retains one random feature among the correlated ones, the selection of parameter λ is a trade-off between the prediction error and L1-norm regularization we used λ = 0.08 in this study.

The prediction model was chosen to depict the correlation between the connectome-based feature and the UPDRS-III score. Considering the size of the dataset, it is not convincing if we only use part of the training-validate-test dataset. Therefore, the Leave-One-Out-Cross-Validation (LOOCV) was used to maximize the loss function (22, 23). In LOOCV, N-1 (N is number of subjects, *N* = 62) samples were used as training data and the remaining samples were used as validation data. The left subjects were used as the input to the training model which was derived with inner training data, generating the estimated UPDRS-III scores. This loop was repeated *N* times to test all subjects. Each time, the predicted UPDRS-III scores for the left-one-out subjects, the identified FCs, and their corresponding weights in the training model were obtained. By pulling all testing subjects across *N* loops together, we obtained the prediction results for all subjects. Thus, there were *N* regression models of the same type with the same parameters for learning and predicting different data. The prediction performance was assessed by the Pearson correlation (with Bonferroni correction) between the model predicted UPDRS-III scores and the actual scores. Permutation test (1,000 times) was carried out to access the significance (24). Mean Absolute Error (MAE) were used to measure the magnitude of the error between the predicted and the actual UPDRS-III scores.

## Feature Identification

Since we applied a cross-validation strategy to estimate the UPDRS-III scores, in each iteration, slightly different connections were selected. The relative weights for all selected connections were determined by averaging the regression weights of all loops. For better interpretation and visualization (14, 15), we grouped the 246 FC nodes into 24 relatively large brain regions defined by the Brainnetome atlas (20), and estimated the inter-region contributing power by averaging the weights of all FCs connecting between two specific macroscale regions.

## Results

### Clinical Data

Firstly, we compared the UPDRS-III scores before and after levodopa therapy (“medication-off” and “medication-on,” respectively). The paired *t*-test shows significant difference (*p* < 0.0001) of the UPDRS-III scores between medication-off (44.1 ± 12.0) and -on (22.2 ± 11.8), which demonstrates the efficacy of levodopa.

### Feature Selection for Medication-off and Medication-on

**Figure 2:**
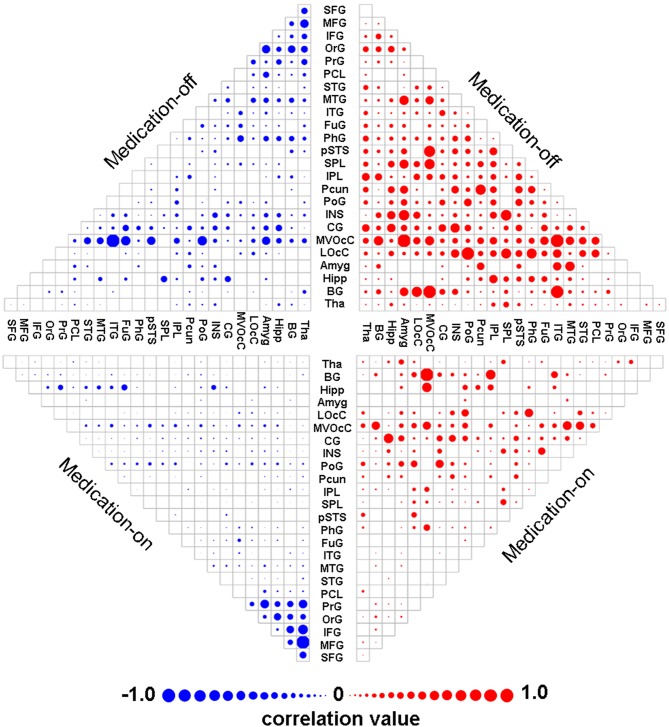
Mean weights distribution of whole-brain dnE network for each of the two states including medication-off and medication-on. The mean contributing weights of whole-brain dnE network connections for medication-off and medication-on were calculated by computing the correlation between connections of each macro-scale with the traits of UPDRS-III. Blue represents negative correlation and red represents positive correlation. As shown in the matrix plot, the 246 FC nodes are grouped into 24 macro-scale brain regions that are anatomically defined by the Brainnetome atlas. For the matrix plots, rows and columns represent predefined macro-scale regions in the Brainnetome Atlas, and a bigger circle represents a higher predictive weight. Names of 24 macroscale regions were colored according to their lobe locations. dnE, dynamic nodal efficiency.

The mean contributing weights of whole-brain dnE network are shown in Figure 2. For the medication-off status, MedioVentral Occipital Cortex (MVOcC), are the important regions. For the medication-on status, the important regions are frontal regions including the Inferior Frontal Gyrus (IFG), Middle Frontal Gyrus (MFG), Superior Frontal Gyrus (SFG) and Orbital Gyrus (OrG).

### Prediction Performance

**Figure 3:**
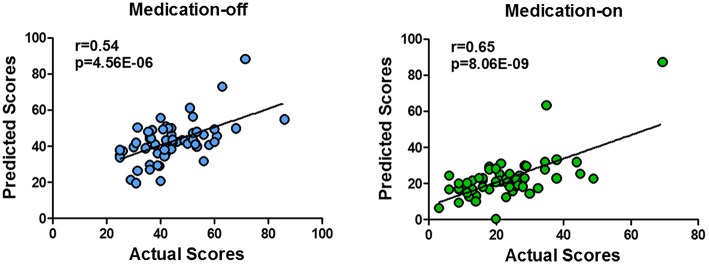
Scatter plot of the predicted four states of the UPDRS-III scores with respect to their true values based on the prediction framework using whole-brain dnE network. With the connectome-based prediction framework, Pearson's correlation of *r* = 0.54 (*p* = 4.56 × 10^−6^) and *r* = 0.65 (*p* = 8.06 × 10^−9^) were achieved for medication-off and medication-on, respectively, in the nested cross-validation using whole-brain dynamic nodal efficiency network. The abbreviations of the brain areas are from the Brainnetome atlas (http://atlas.brainnetome.org/) (20).

The dnE network based prediction models achieved significant correlation between the predicted and the true UPDRS-III scores of either medication-off or -on for the 62 PD patients (Figure 3). Specifically, Pearson correlation of *r* = 0.54 (*p* = 4.56 × 10^−6^, MAE = 9.49) and *r* = 0.65 (*p* = 8.06 × 10^−9^, MAE = 7.52) were obtained for medication-off and –on, respectively. All the results passed Bonferroni corrections for the multiple comparisons. The *p*-value of the permutation test is 0.004 and 0.001 for medication-off and –on, respectively.

### Connections Identified

**Figure 4:**
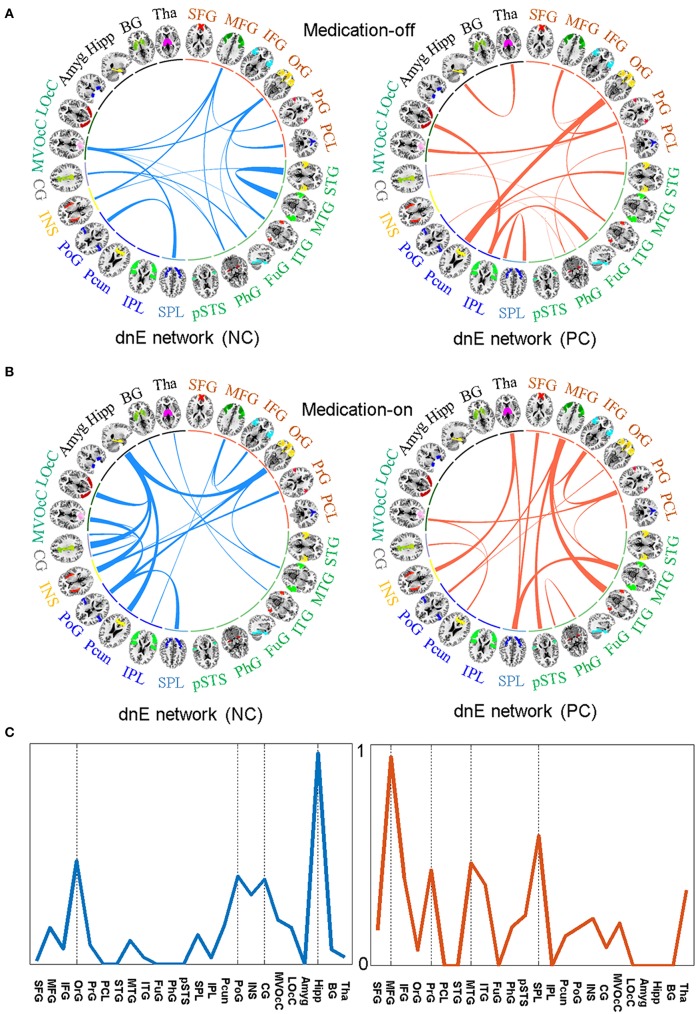
The identified features in the dnE network between medication-off **(A)** and medication-on **(B)**, respectively, including the negative connections (NC) represented by blue and the positive connections (PC) represented by red, respectively. The width of the inter-node lines represents the strength of connections. For the negative connections, stronger connectivity reflects smaller disease severity and better recovery after drug therapy. The positive connection case is on the contrary. The prediction efficacy of each node for medication-on is shown in **(C)**. The results are from the regression model and are normalized to the range of 0 to 1. As shown in the circle plots, the 246 FC nodes (inner circle) are grouped into 24 macro-scale brain regions (outer brain representations) that are anatomically defined by the Brainnetome atlas. Specifically, nodes incorporated in each of 24 macro-scale brain areas are plotted with different colors, which delineate their corresponding anatomy locations in the outer brain representations.

The relative weights of dnE network connecting between each pair of the 24 anatomically defined macro-scale areas are displayed in Figure 4. The identified features in the dnE network of either medication-off or -on include the negative connections (blue) and positive connections (red). The width of the inter-node lines represents the strength of the connections. For the negative connections, stronger connectivity reflects lower disease severity thus better recovery after drug therapy. The positive connection case reflects the contrary. Specifically, for medication-off, dnE network connections show more contributing power between Middle Temporal Gyrus (MTG) and STG, Postcentral Gyrus (PoG) and Superior Parietal Lobule (25). The stronger the connections, the better the recovery of PD. There is a negative correlation between the strength of some connections and the UPDRS-III scores, such as the connections between Precuneus (Pcun) and Orbital Gyrus (OrG), Inferior Parietal Lobule (IPL), and lateral Occipital Cortex (LOcC), IPL, and Fusiform Gyrus (FuG). The stronger the connections, the worse the recovery of PD.

For medication-on, in terms of the feature analysis of predicting recovery effect after the drug therapy, the contributing power is mainly concentrated on hippocampus (Hipp), PoG, Pcun, Cingulate Gyrus (CG), Insular Gyrus (INS), and OrG. Particularly, the features of the connections between Hipp and CG, INS, Pcun, OrG, respectively, have much influence on the recovery after therapy. The stronger the connections, the better the recovery. For other regions, such as MFG and MTG, the stronger the connection between MFG and Inferior Temporal Gyrus (ITG) or the connection between MTG and SPL, the worse the recovery of PD. The prediction efficacy of each region is shown in Figure 4C. The results are from the regression model and are normalized to the range of 0 to 1.

## Discussion

Through analyzing the efficiency of correlation networks using rs-fMRI, the present study investigated the important features of connections that are correlated to the post-therapy disease severity (UPDRS-III scores) after taking levodopa in the 62 PD patients. Studying the effect of drug therapy is an important topic in PD research. Finding what influences the effect of drug therapy is of great significance. This work predicted the correlation between the dnE network and the actual effect of drug therapy by training a regression model. The major findings of the present study are as follows: (1) The connection efficiency of networks based on rs-fMRI can effectively depict the severity of PD (UPDRS-III scores), and further predict the recovery effect after drug therapy. (2) The Hipp region is an important area that indicates drug therapy effect in the dnE network. (3) Increased cortical functional connectivity from ITG and MTG has a negative effect on the recovery. As such, these findings provide new evidence that the rs-fMRI network connectivity strengthening or weakening within key functional networks in dnE network plays an important pathophysiological role in the recovery of PD patients.

The FC analysis of the brain network has revealed that the brain is organized according to a highly efficient small-world topology, combining a high level of segregation (local efficiency) with a high level of global integration (global efficiency) (26). Most of the previous studies did not consider the important dynamic properties of FC, as functional connectivity was assumed to be constant during rs-fMRI scanning. However, dynamic FC may yield novel insights into brain function and dysfunction (8). The sliding window approach is commonly used for examining dynamics in resting-state FC, resulting in quantification of the time-varying behavior of a chosen metric. In this study, we selected the nodal global efficiency (i.e., nodal efficiency) as the metric, then obtained the time-varying behavior of each nodal global efficiency. Global efficiency is a network attribute that quantifies how easily information can be exchanged over the network. It provides information on the communication efficiency of a network as a whole, with higher values indicating more efficient information transmission through the whole brain. We further calculated the inter-node correlation of the dnE, to further reveal the synchronization of the dynamic property of FC between two regions.

The prediction model analyses demonstrated that some specific subnetworks with decreased connectivity are correlated with the recovery effect after drug therapy. The regions mainly include parietal lobe, insular lobe, limbic lobe, and Hipp. The connections between these regions are directly positively correlated to the recovery after drug therapy: the stronger the connections, the better the recovery (lower UPDRS-III scores). Nonetheless, there are several key pathways in the SPL, ITG, MFG, MTG showed negative influence on the recovery of PD.

Previous studies showed that the decreased functional connectivity of the temporal cortex is related to the disease progression of PD (27, 28). We drew the similar conclusion here, especially for the connection between MTG and STG. In addition, we found that a distributed set of regions in the frontal lobe, temporal lobe, occipital lobe, and parietal lobe showed decreased inter-node correlation of dnE in these patients. The decreased correlation is related to the high post-therapy UPDRS-III scores of PD patients. It reveals that these regions are also related to the disease progression of PD. Among these important connections exist, such as the connection between the paracentral cortex and OrG. Some other studies have also reported that connections among these regions is related to cognitive decline (29).

This study shows that the Hipp region, which was previously reported to influence dementia (30), is also an important area to indicate drug therapy effect. Previous study also shows that there is a high correlation between PD and dementia, i.e., PD with dementia (31). This study reveals that individualized recovery effect after drug therapy can be influenced by the functional connections between Hipp and other areas, on which enough attention should be paid before therapy.

The selected features (Figure 2) are not necessarily useful for the following prediction. For example, the frontal area appears to be important in the feature selection, but the Hipp area is the dominant feature for prediction. Therefore, how to improve feature selection needs to be further studied. Reducing feature dimension while maintaining all of the connection information might need to be carefully considered.

There are several limitations of this study. First, the number of subjects was relatively small to draw firm conclusions. Second, the dynamic property of FC we investigated in this study is only the commonly used global efficiency. It should be noted that choosing the global efficiency does not mean less importance of other network properties. Other dynamic properties, such as local efficiency, may provide additional information and are worthwhile to be further investigated in future studies. Third, the long-term follow-up study should be carried out in the future to follow the outcome of patients.

## Conclusion

In this study we studied a sample of 62 PD patients and calculated the dnE based on rs-fMRI. With connectome-based predictive models using LASSO, we demonstrated that the dnE properties can successfully predict the post-therapy severity level of PD patients after taking levodopa. The contributed regions for the prediction include hippocampus, post-central gyrus, cingulate gyrus, and orbital gyrus. Specifically, the connections between hippocampus and cingulate gyrus, hippocampus and insular gyrus, insular gyrus and orbital gyrus are positively related to the recovery after drug therapy. The analysis of these connectivity features can provide guidance information for clinical therapy in PD patients.

## Ethics Statement

This study was carried out in accordance with The Nuremberg Code, World Medical Association Declaration of Helsinki and International Ethical Guidelines for Biomedical, Ethics Committee of Tsinghua University Yuquan Hospital. Research Involving Human Subjects, and with written informed consent from all subjects. All subjects gave written informed consent in accordance with the Declaration of Helsinki. The protocol was approved by the Ethics Committee of Tsinghua University Yuquan Hospital.

## Author Contributions

XL contributed to the experiments, data analysis, and writing of the manuscript. YX and SL contributed to performing the experiments and revising the manuscript. RZ and LH contributed to the data collection. ZH and YT revised the manuscript. ZN contributed to the manuscript revision. YM and HG are the guarantors of this study and had complete access to all data in the study. All authors are accountable for the contents of this research.

### Conflict of Interest Statement

The authors declare that the research was conducted in the absence of any commercial or financial relationships that could be construed as a potential conflict of interest.
